# Soluble interleukin-18 receptor complex is a novel biomarker in rheumatoid arthritis

**DOI:** 10.1186/ar3295

**Published:** 2011-03-24

**Authors:** Satoko Takei, Tomoaki Hoshino, Kazuko Matsunaga, Yuki Sakazaki, Masanori Sawada, Hanako Oda, Shin-ichi Takenaka, Haruki Imaoka, Takashi Kinoshita, Seiyo Honda, Hiroaki Ida, Taka-aki Fukuda, Hisamichi Aizawa

**Affiliations:** 1 Division of Respirology, Neurology, and Rheumatology, Department of Medicine, Kurume University School of Medicine, 67 Asahi-machi, Kurume, Fukuoka 830-0011, Japan

## Abstract

**Introduction:**

There has been no report in the literature of a soluble form of interleukin (IL)-18 receptor α (IL-18Rα). In this study, we evaluated the levels and characteristics of soluble IL-18Rα (sIL-18Rα) in the sera of patients with rheumatoid arthritis (RA) and compared these results to control populations.

****Methods**:**

The sIL-18Rα complex was isolated from pooled human blood serum using an anti-IL-18Rα monoclonal antibody affinity column. The purified sIL-18Rα was then examined using Western blot analysis and used in experiments to evaluate the effects on an IL-18-responsive natural killer (NK) human cell line, NK0. An enzyme-linked immunosorbent assay was developed, and sera from 145 patients with RA, 6 patients with adult-onset Still's disease, 31 patients with osteoarthritis (OA), 39 patients with systemic lupus erythematosus (SLE) and 67 controls were tested, along with levels of immunoglobulin M, rheumatoid factor, anticyclic citrullinated peptide antibody, IL-18, IL-13 and interferon (IFN)-γ. Area under the receiver operating characteristic curve (ROC-AUC) analysis was used to evaluate the diagnostic utility of the sIL-18Rα complex.

**Results:**

The isolated sIL-18Rα complex can be associated with IL-18 and the soluble form of the IL-18Rβ chain. The sIL-18Rα complex bound to the surface to the NK0 cell line, antagonized the stimulatory effects of IL-18 and IL-2 on the NK0 cell line and inhibited IFN-γ production by the cells. The serum levels of sIL-18Rα complex in RA (186.0 ± 33.5 ng/mL, *n *= 145) and adult-onset Still's disease (98.2 ± 8.9 ng/mL, *n *= 6) were significantly (*P *< 0.001) higher than those in the healthy controls (52.3 ± 8.5 ng/mL, *n *= 67), OA (38.6 ± 5.4 ng/mL, *n *= 31), SLE (44.6 ± 3.2 ng/mL, *n *= 39). The serum level of sIL-18Rα complex was not significantly different between RA and adult-onset Still's disease patients. The serum levels of IL-18, IL-13 and IFN-γ in the RA patients were significantly (*P *< 0.01) higher than in OA and SLE patients as well as healthy controls. ROC-AUC analysis of the serum concentration of sIL-18Rα indicated that it was significantly diagnostic of RA. Moreover, a tumor necrosis factor inhibitor, etanercept, significantly (*P *< 0.0001) decreased levels of sIL-18Rα in the sera of 29 RA patients 6 months after treatment.

**Conclusions:**

The sIL-18Rα complex could be a potentially useful biomarker for the diagnosis of RA.

## Introduction

Interleukin (IL)-1α and IL-1β have two homologous receptors IL-1 receptor 1, type I (IL-1RI) and IL-1R, type II (IL-1RII). Functional IL-1R is a complex comprising IL-1RI and IL-1 receptor accessory protein (IL-1RAcP) (see reviews in [1-3]). Upon binding of IL-1α or IL-1β, IL-1RI forms a complex with IL-1RAcP and initiates a cytosolic signaling cascade (myeloid differentiation primary response protein (MyD88), IL1R-associated kinase (IRAK) and tumor necrosis factor-associated factor 6 protein (TRAF6), as well as activation of nuclear factor κB (NFκB), c-Jun N-terminal kinase (JNK) and p38 mitogen-activated protein kinase (p38 MAPK)). In contrast, 60-kDa IL-1RII functions as a nonsignaling "decoy" receptor. It has been reported that IL-1RI has a soluble form (sIL-1RI) [4], which can be generated by metalloprotease [5]. Soluble IL-1RII (sIL-1RII) is also generated primarily by proteolytic cleavage in response to a variety of stimuli and can attenuate excessive IL-1 bioactivity by preferentially binding IL-1β. In contrast, soluble IL-1RAcP (sIL-1RAcP) is generated by alternative splicing rather than by ectodomain cleavage. The soluble receptors sIL-1RI, sIL-1RII and sIL-RAcP may attenuate IL-1 signaling [5].

IL-18, a member of the IL-1 family, is well-known to play an important role in T helper 1 (Th1) cell polarization. IL-18 can also act as a cofactor for T helper 2 (Th2) cell development and immunoglobulin (Ig) E production [6-9]. Many lines of evidence indicate that IL-18 plays an important role in the pathogenesis of inflammatory diseases of the bowel, heart and lung [8-12]. The IL-18R belongs to the IL-1R family [1], and the IL-18R complex is composed of the IL-18Rα and IL-18Rβ chains. IL-18Rα (IL-1R5 or IL-1R-related protein 1 (IL-1Rrp1)) is the extracellular signaling domain [1,13], whereas IL-18Rβ (IL-R7 and accessory protein-like (AcPL) or IL-18R accessory protein (IL-18RAP)) is an adapter molecule [1] in the complex. IL-18Rα alone binds IL-18 with low affinity (dissociation constant (*K*_d_) 20 to 50 nM), and IL-18Rβ alone cannot bind IL-18. However, IL-18Rα can bind with high affinity (*K*_d _0.3 nM) by recruiting IL-18Rβ. Upon IL-18's binding to the IL-18R complex, IL-18 signaling uses the same adapter molecules (MyD88, TRAF6 and IRAK) as the IL-1 family cytokines and elicits similar responses (activation of NFκB, JNK and p38 MAPK) [1]. Both the IL-18Rα and IL-18Rβ chains are thought to be essential for IL-18-mediated signaling [14,15]. IL-18 binding protein (IL-18BP) is a soluble protein that binds to IL-18 with high affinity (*K*_d _0.4 nM) and exerts neutralizing activity against IL-18 [1,8,16]. Another member of the IL-1 family, IL-33, binds to the IL-1R family ST2 (IL-33Rα) and IL-1RAcP (IL-33Rβ) complex on cell surfaces and induces the production of Th2 cytokines such as IL-4, IL-5 and IL-13 [3]. Soluble ST2 functions as a soluble decoy receptor for IL-33 and blocks IL-33 signaling [17]. These characteristics suggest that the IL-18Rα or IL-18β chain may have a soluble form.

Recently, the cytokine milieu of the joint in RA patients has become well-understood, and data from human clinical trials are now available. Therapies designed to block the effects of inflammatory cytokine tumor necrosis factor (TNF)-α and the action of the IL-6 receptor (IL-6R) are well-known to be effective in many RA patients. Rheumatoid subcutaneous nodules have the features of Th1 granulomas, with abundant expression of inflammatory cytokines, including interferon (IFN)-γ and IL-18 [8]. The level of IL-18 is reportedly increased in both the serum and rheumatoid synovial fluid, as well as in the bone marrow, of patients with RA, juvenile RA, adult-onset Still's disease and psoriatic arthritis [8,18,19]. Moreover, recombinant human IL-18 (rhIL-18) is being actively investigated for its potential efficacy and safety in the treatment of RA. Recent data illustrate the importance of IL-18 in the induction and perpetuation of chronic inflammation in RA patients [9,19].

As yet, no reported study has focused on the sIL-18Rα chain. In the present study, we attempted to isolate and characterize the human sIL-18Rα complex from human serum. We also found that serum levels of the complex in RA patients were significantly higher than those in healthy controls. Our findings suggest that the sIL-18Rα complex could be a potentially useful biomarker for the diagnosis of RA.

## Materials and methods

### Subjects

One-hundred forty-five patients (24 males and 121 females) diagnosed with RA were studied. The diagnosis of RA was based on the criteria of the American College of Rheumatology (ACR) [20]. Joint damage was assessed by a radiologist on the basis of the Steinbrocker global score (classes I to IV). The Steinbrocker functional classification was used by the physician to rate the extent of physical disability on a four-point scale ranging from class I, "complete functional capacity to carry out all usual duties without handicap," to class IV, "largely or wholly incapacitated, and bedridden or confined to a wheelchair" [21]. The rheumatoid arthritis 28-joint Disease Activity Score (DAS28) based on C-reactive protein (CRP) level and the DAS28 based on erythrocyte sedimentation rate (ESR), as well as the Health Assessment Questionnaire (HAQ) score, were calculated as previously reported [22,23]. All patients had been consecutively monitored from 2005 to 2010 at Kurume University Hospital (Kurume, Japan). Serum samples were also obtained from 67 age-matched healthy volunteers, who served as controls. Laboratory data, including white blood cell (WBC) count, CRP level, IgM rheumatoid factor (RF) and anticyclic citrullinated peptide (CCP) antibody levels were examined at Kurume University Hospital as reported previously [24]. Six patients with adult-onset Still's disease were diagnosed according to criteria published by Yamaguchi *et al*. [25]. Thirty-one patients with osteoarthritis (OA), and 39 patients with systemic lupus erythematosus (SLE) were diagnosed as previously reported [26,27]. The details of these subjects are shown in Table 1. Sample collection and all procedures were approved by the ethics committee of Kurume University in accordance with the ethical standards of the Helsinki Declaration of 1975. Informed consent was obtained from all patients and healthy volunteers.

**Table 1:** Characteristics of rheumatoid arthritis (RA) patients and control participants^a^

Participant characteristics	Control (*n *= 67)	RA (*n *= 145)	AOSD (*n *= 6)	OA (*n *= 31)	SLE (*n *= 39)
Mean age, yr (± SEM)	60.7 ± 2.1	56.7 ± 1.2	54.3 ± 9.9	76.3 ± 1.5^b^	38.8 ± 2.1^b^
Sex, *n*					
Male	40	24^b^	1^b^	6^b^	6^b^
Female	27	121	5	25	33
Smoking status, *n*					
Nonsmoker	37	32	1	0	0
Smoker	30	12	0	0	0
Unknown	0	101	5	31	39
Joint damage^c^, *n*					
I	ND	42	ND	ND	ND
II	ND	47	ND	ND	ND
III	ND	20	ND	ND	ND
IV	ND	36	ND	ND	ND
Functional classification^d^, *n*					
I	ND	24	ND	ND	ND
II	ND	92	ND	ND	ND
III	ND	29	ND	ND	ND
IV	ND	0	ND	ND	ND
Mean DAS28 (CRP) (± SEM)	ND	4.3 ± 0.1	ND	ND	ND
Mean DAS28 (ESR) (± SEM)	ND	4.9 ± 0.1	ND	ND	ND
Mean HAQ (± SEM)	ND	5.8 ± 0.7	ND	ND	ND
Mean WBC (cell count/μL) (± SEM)	ND	7,307.3 ± 211.0	8,950.0 ± 533.1	5,134.7 ± 304.8	ND
Mean CRP level, mg/dL (± SEM)	ND	2.27 ± 0.22	2.15 ± 1.14	0.08 ± 0.02	ND
Mean ESR, mm/hour (± SEM)	ND	45.0 ± 2.7	40.3 ± 18.6	ND	ND
RF-positive^d^, *n* (%)	2/64 (3.1%)	106/145 (73.1%)	0/6 (0%)	2/30 (6.7%)	2/39 (5.1%)
Mean RF, U/mL (± SEM)	3.7 ± 1.3	158.0 ± 20.9 ^b^	4.8 ± 2.7	5.4 ± 1.3^b^	3.8 ± 1.0
CCP-positive^e^, *n *(%)	0/59 (0%)	121/145 (83.4%)	1/6 (16.7%)	0/30 (0%)	3/39 (7.7%)
Mean CCP, U/mL (± SEM)		0.3 ± 0.1	261.5 ± 27.2^b^	22.5 ± 22.3	0.5 ± 0.1^b^
PSL, *n *(%)	ND	89/145 (61.4%)	ND	ND	ND
Mean dose, mg/day (± SEM)	ND	5.91 ± 0.32	ND	ND	ND
MTX, *n *(%)	ND	58/145 (40.0%)	ND	ND	ND
Mean dose, mg/week (± SEM)	ND	6.87 ± 0.34	ND	ND	ND
DMARDs^f^, *n *(%)	ND	117/145 (80.7%)	ND	ND	ND

^a^RA = rheumatoid arthritis; AOSD = adult-onset Still's disease; OA = osteoarthritis; SLE = systemic lupus erythematosus; DAS28 = 28-joint Disease Activity Score; HAQ = Health Assessment Questionnaire; WBC = white blood cell; CRP = C-reactive protein; ESR = erythrocyte sedimentation rate; RF = rheumatoid factor; CCP = anticyclic citrullinated peptide antibody; PSL = prednisolone; MTX = methotrexate; DMARDs = disease-modifying antirheumatic drugs; ^b^*P *< 0.05 vs. healthy controls; ^c^Steinbrocker global score (I to IV) [21]; ^c^Steinbrocker functional classification was used, ranging from class I, "complete functional capacity to carry out all usual duties without handicaps," to class IV, "largely or wholly incapacitated with (the person) bedridden or confined to wheelchair" [21]; ^d^cutoff serum concentration of the immunoglobulin M rheumatoid factor (RF) is 20 units/mL; ^e^cutoff serum concentration of the anti-CCP antibody is 4.5 U/mL; ^f^none of RA patients were treated with tumor necrosis factor α inhibitor at this point.

### Reagents

rhIL-18 (catalog no. B003-5) was purchased from MBL (Nagoya, Japan). rhIL-18Rα (IL-1 R5) or Fc chimera (catalog no. 816-LR), mouse antihuman IL-18Rα monoclonal antibody (mAb) (clone 70625; catalog no. MAB840) and mouse antihuman IL-18Rβ mAb (clone 132016, catalog no. MAB1181; and clone 132029, catalog no. MAB118) were purchased from R&D Systems, Inc. (Minneapolis, MN, USA). Rabbit antihuman IL-18Rα polyclonal antibody (pAb) [28] was kindly provided by Dr Tsukasa Seya (Hokkaido University, Sapporo, Japan). Antihuman-IL-18 mAb (clone 8 (IgG2a)) was kindly provided by Dr Do-Young Yoon (Laboratory of Cellular Biology, Korea Research Institute of Bioscience and Biotechnology, Taejon, Korea [29]). Antihuman-IL-18Rα mAb (H44 (IgG1)) was established in our laboratory [30] and is commercially available from BD Pharmingen (San Diego, CA, USA), eBioscience (San Diego, CA, USA), BioLegend (San Diego, CA, USA) and Serotec (Oxford, UK).

### Purification of soluble human interleukin-18 receptor α complex from human blood serum

Mouse antihuman IL-18Rα mAb H44 was used on an anti-IL-18Rα mAb affinity column to isolate the complex. The H44 hybridoma cell line was cultured in serum-free medium (GIT; Wako Pure Chemical Industries, Ltd., Osaka, Japan). Proteins in cell-free culture supernatants were precipitated with ammonium sulfate and then further purified using a protein G column (GE Healthcare, Tokyo, Japan) as reported previously [31]. This purified H44 mAb was coupled to a HiTrap NHS-activated HP column (GE Healthcare) in accordance with the manufacturer's protocol. Samples of pooled human blood serum were then applied to this affinity column. Phosphate buffer (10 mM, pH 6.8) was used as the binding buffer, 10 mM phosphate buffer plus 50 mM NaCl (pH 6.8) was used as the washing buffer, 100 mM glycine buffer (pH 2.5) was used as the elution buffer and 1 M phosphate buffer (pH 8.0) was used as the neutralization buffer. All buffers were filtered through a 0.45-μm filter (Millipore, Tokyo, Japan) before the experiments. Serum samples were diluted twofold with the binding buffer, filtered through a 0.45-μm filter (Millipore) and applied to an antihuman IL-18Rα mAb affinity column that had been equilibrated beforehand with the binding buffer. The affinity column was washed with the washing buffer. The elution buffer was then allowed to flow through the column, and every 1-mL sample was collected into a test tube containing 50 μL of neutralization buffer (fractions collected were denoted in numerical order as fractions 1, 2, 3 and so on), with the fractions being monitored with UV radiation at 280 nm. The collected fractions were dialyzed against distilled H_2_O (Otsuka Pharmaceutical Co., Ltd., Tokushima, Japan) in the presence of 1 mM phenylmethanesulfonyl fluoride (Sigma) at 4°C. Purified hIL-18Rα complex was concentrated using Centricon centrifugal filters (Millipore), and all of the samples and buffers were then kept at 4°C.

### Establishment of enzyme-linked immunosorbent assay system for measurement of serum soluble human interleukin-18 receptor α complex

H44 mouse antihuman IL-18Rα primary mAb dissolved at 4 μg/mL in phosphate-buffered saline (PBS) was dispensed into enzyme-linked immunosorbent assay (ELISA) plates (Nunc, Tokyo, Japan) in aliquots of 100 μL/well and left undisturbed overnight at 4°C to allow it to become immobilized. The plates were then washed twice with 200 μL of PBS buffer containing 0.5% Tween 20, and 200 μL/well of 10% Block Ace blocking solution (Dainippon Sumitomo Pharma Co., Ltd., Osaka, Japan) were added and left for at least 2 hours at room temperature to prevent nonspecific adhesion of the secondary antibody to the plates. The plates were then washed again twice with 200 μL of PBS buffer containing 0.5% Tween 20. Human blood serum samples were then aliquoted at 100 μL/well. rhIL-18Rα protein (R&D Systems, Inc.) diluted to 400, 200, 100, 50, 25, 12.5 and 6.25 ng/mL was used as the standard for the human sIL-18Rα complex ELISA system. After 2 hours of incubation at room temperature, each well was washed three times with 200 μl of PBS buffer containing 0.5% Tween 20. Next, 2 μg/mL biotin-labeled rabbit antihuman IL-18Rα secondary pAb was dispensed at 100 μL/well, followed by incubation for 90 minutes at room temperature, and then each well was washed four times with 200 μl of PBS buffer containing 0.5% Tween 20. This was followed by addition of 100 μL of 0.5 μg/mL streptavidin-bound horseradish peroxidase (Upstate, Tokyo, Japan) to each well, and the plates were left undisturbed for 30 minutes at room temperature. Each well was then washed five times with 200 μL of PBS buffer containing 0.5% Tween 20. 2,2'-Azinobis [3-ethylbenzothiazoline-6-sulfonic acid]-diammonium salt (ELISA POD Substrate A.B.T.S. kit; Nakarai, Kyoto, Japan) was then added at 100 μl/well, and the plates were left undisturbed for 30 minutes at room temperature, followed by addition of a stop solution at 100 μL/well to stop the enzyme reaction. The amounts of the sIL-18Rα complex protein were determined by measuring the absorbance at 450 nm in comparison with the standard rhIL-18Rα protein sample. The limit of sensitivity of this ELISA system was <5 ng/mL.

### Sodium dodecyl sulfate-polyacrylamide gel electrophoresis and Western blot analysis

SDS-polyacrylamide gel electrophoresis (PAGE) was performed using premade 10% to 20% or 15% to 25% polyacrylamide gel (Multigel II Mini; Dai-ichi Kagaku Yakuhin, Tokyo, Japan). The gel was stained with Coomassie Brilliant Blue (CBB) or assessed using Western blot analysis as described in our previous reports [30,32]. MagicMark™ Western Standard (Invitrogen, Carlsbad, CA, USA) was used for protein band standardization.

### Cytokine enzyme-linked immunosorbent assays

Levels of mature IL-18, IL-13 and IFN-γ in serum were measured using ELISA kits (IL-18: MBL, Nagoya, Japan; IL-13 and IFN-γ: R&D Systems, Inc.). The limits of sensitivity of these ELISA kits were 12.5 pg/mL, 32 pg/mL and 8 pg/mL, respectively.

### Flow cytometric analysis

The purified sIL-18Rα complex protein and H44 mAb were labeled with biotin as reported previously [31]. Cells were incubated with 2 μg of biotinylated control mouse IgG1 (Caltag, Burlingame, CA, USA), 2 μg of biotinylated anti-IL-18Rα mAb H44 (mouse IgG1) or 10 μg of biotinylated anti-IL-18Rα complex protein at 4°C, then washed with PBS and incubated with streptavidin-PE (BD Pharmingen), followed by flow cytometric analysis as reported previously [12].

### Interferon-γ production inhibition assay using a human interleukin-18-responsive natural killer cell line

We used the hIL-18-responsive human natural killer (NK) cell line NK0, a subclone of the original NK92 cell line. Using NK0 cells, we performed IFN-γ production inhibition assay *in vitro *as reported previously [29,30]. Briefly, NK0 cells were washed three times and cultured in RPMI 1640 medium with 10% fetal calf serum (FCS) for 18 hours. The cells were then washed once, suspended at 5 × 10^5 ^cells/mL in RPMI 1640 medium with 10% FCS and pretreated with sIL-18Rα complex (0.01 to 30 μg/mL) for 10 minutes at room temperature. The cells were then treated with rhIL-2 (50 IU/mL) plus rhIL-18 (50 ng/mL) for 18 hours. Production of human IFN-γ in the supernatants was analyzed using ELISA kits (R&D Systems, Inc.).

### Statistical analysis

Data are presented as means ± standard errors of the mean (SEM). Differences between the RA patients and the controls were analyzed by using the Wilcoxon rank-sum test. The sensitivity and specificity for the diagnosis of RA were analyzed using area under the receiver operating characteristic curve (ROC-AUC) analysis generated by logistic regression as reported previously [33]. Statistical analysis was performed using the JMP 7.0.1 software package (SAS Institute, Japan, Tokyo, Japan). Differences were considered significant at *P *< 0.05.

## Results

### Isolation and characterization of soluble human interleukin-18 receptor α complex from human sera

**Figure 1:**
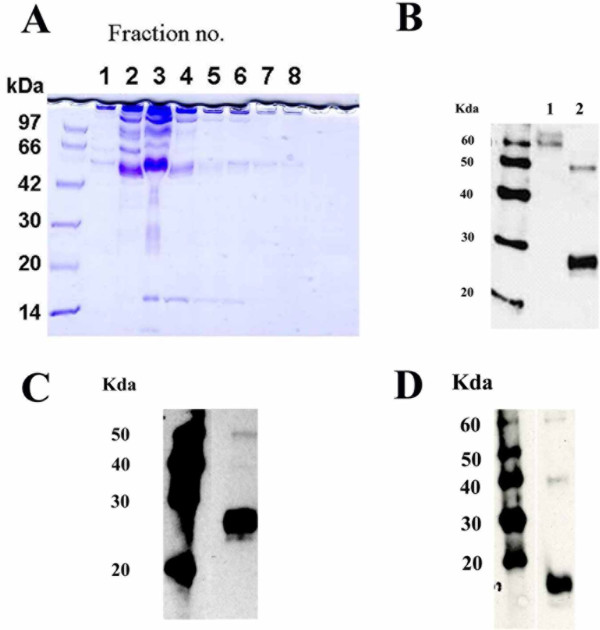
**Soluble interleukin-18 receptor α complex is associated with interleukin-18 and the soluble form of the interleukin-18 receptor β chain**. **(A) **Sodium dodecyl sulfate (SDS)-polyacrylamide gel electrophoresis of the serum interleukin (IL)-18 receptor α (IL-18Rα) complex. Purified H44 monoclonal antibody (mAb) was coupled to a 5-mL HiTrap NHS-activated HP column (GE Healthcare). Pooled human blood serum (120 mL) was applied to this affinity column. The IL-18Rα complex was eluted with elution buffer at a flow rate of 2 mL/minute. Every 1 mL of the elution buffer was collected into a test tube containing 50 mL of neutralization buffer (collected fractions were denoted in order as fractions 1, 2, 3 and so on). A 10-μL aliquot of every fraction (fractions 1 to 8) was treated with the same volume of sample buffer containing 4% SDS (Tris-Glycine SDS Sample Buffer (2×); Invitrogen). Electrophoresis was carried out in the presence of 0.1% SDS, and the gel was stained with Coomassie Brilliant Blue. **(B) **Western blots of the serum IL-18Rα complex using an antihuman IL-18Rα mAb are shown. Western blot analysis was performed using antihuman IL-18Rα mAb 70625 (R&D Systems, Inc.). Lane 1: Western blot showing 1 μg of rhIL-18Rα/Fc chimera protein (R&D Systems, Inc.). Lane 2: Western blot showing 5 μg of isolated serum IL-18Rα complex. **(C) **Western blot showing serum IL-18Rα complex using an antihuman IL-18 mAb. Western blot analysis was performed using antihuman IL-18 mAb clone 8 with 5 μg of isolated serum IL-18Rα complex. **(D) **Western blot showing serum IL-18Rα complex using an antihuman IL-18Rβ mAb. Western blot analysis was performed using antihuman IL-18Rβ mAb 132016 (R&D Systems, Inc.) with 5 μg of isolated serum IL-18Rα complex.

To determine whether the human sIL-18Rα complex is present in the sera of healthy individuals, we prepared an anti-IL-18Rα mAb affinity column to isolate the complex using mouse antihuman IL-18Rα H44 mAb [30]. Pooled samples of human blood serum were applied to this affinity column as described in Materials and methods. The elution buffer was allowed to flow through the column and was collected in 1-mL fractions. We then measured the levels of sIL-18Rα complex in these fractions using a newly developed sandwich ELISA system. The concentrations of sIL-18Rα complex in these fractions were <100, 1,797, 1,778, 1,259, 293, <100, <100 and <100 ng/mL, respectively. SDS-PAGE followed by CBB staining showed that the isolated sIL-18Rα complex exhibited one major band of approximately 50 kDa and other bands of approximately 60 to 80, 30 and 15 kDa (Figure 1A).

We performed Western blot analysis to confirm that the IL-18Rα protein was present in fraction 3. This step showed that anti-IL-18Rα mAb clone 70625 detected a band of approximately 60 kDa among the rhIL-18Rα/Fc chimera proteins (lane 1 in Figure 1B). The antibody detected bands of approximately 50 and 30 kDa in the isolated sIL-18Rα complex (lane 2 in Figure 1B), suggesting that the complex had at least two different sIL-18Rα proteins. Upon IL-18 binding, the IL-18Rα chain forms a complex with the IL-18Rβ chain on cell surfaces [1]. Therefore, we hypothesized that the sIL-18Rα complex associates with IL-18 and sIL-18Rβ proteins, and we investigated this possibility using Western blot analysis. The anti-IL-18 mAb (clone 8) detected a strong band of approximately 30 kDa and a weak band of 50 kDa (Figure 1C). As the IL-18 monomer has a calculated molecular mass of 14 kDa [34], IL-18 associated with the IL-18Rα complex could be dimeric. The anti-IL-18Rβ mAb (clone 132016) detected bands of approximately 60, 40 and 18 kDa in the isolated sIL-18Rα complex (Figure 1D). Furthermore, a hIL-18 ELISA kit detected IL-18 protein in the isolated sIL-18Rα complex (data not shown). These results suggest that the human sIL-18Rα complex might be associated with dimeric IL-18 and the sIL-18Rβ chain.

### Ability of the soluble interleukin-18 receptor α complex to bind to the surface of a natural killer cell line

**Figure 2:**
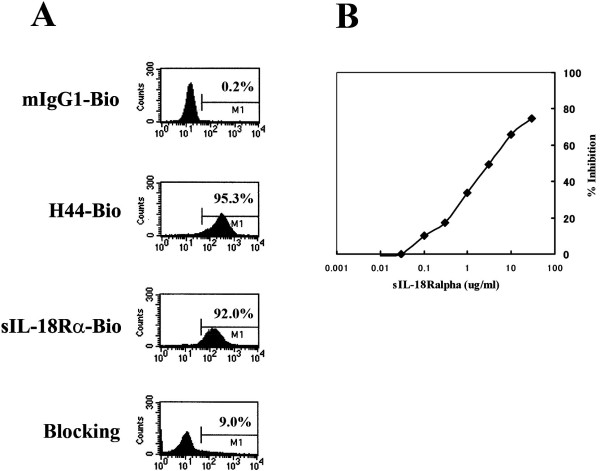
**Binding of soluble interleukin-18 receptor α complex to human natural killer cells expressing interleukin-18 receptor α**. **(A) **The reaction of the human interleukin (IL)-18 receptor α (hIL-18Rα) complex with the human natural killer (NK) cell line NK0 is shown. NK0 cells were stained with control biotin-labeled mouse immunoglobulin G1 (mIgG1-Bio), biotin-labeled anti-IL-18Rα monoclonal antibody clone 44 (H44-Bio) or biotin-labeled hIL-18Rα complex protein at 4°C for 30 minutes. For blocking assays (bottom), excess nonlabeled IL-18Rα complex proteins (80 μg) were preincubated with the cells at room temperature for 30 minutes. The cells were then stained with biotin hIL-18Rα complex at 4°C for 30 minutes. Streptavidin-PE was used for the second-step staining. FACS analysis was performed as described in Materials and methods. Percentages of immunopositive cells are shown in each graph. sIL-18Rα-Bio, biotin-labeled soluble interleukin-18 receptor α complex. **(B) **The neutralizing activity of hIL-18Rα complex protein against recombinant hIL-18 (rhIL-18) protein is shown. NK0 cells (5 × 10^5 ^cells/mL) were suspended in RPMI 1640 medium with 10% fetal calf serum and then pretreated with hIL-18Rα complex protein (0.003 to 10 μg/mL) for 15 minutes at room temperature. The cells were then stimulated with rhIL-2 (rhIL-2) (50 IU/mL) plus rhIL-18 (50 ng/mL) for 18 hours. Human interferon-γ in the supernatants was then analyzed using an enzyme-linked immunosorbent assay kit (R&D Systems, Inc.).

We investigated whether the sIL-18Rα complex could bind to NK0 cells, a cell line that strongly expresses the hIL-18Rα and IL-Rβ chains [30,35]. NK0 cells were incubated with 2 μg of biotinylated control mouse IgG1, 2 μg of biotinylated anti-IL-18Rα mAb H44 (mouse IgG1), or 10 μg of biotinylated anti-IL-18Rα complex protein. Figure 2A shows the representative staining patterns of NK0 cells. H44 mAb, but not control IgG1, reacted strongly with NK0 cells as reported previously [30]. sIL-18Rα complex protein also reacted strongly with NK0 cells. However, preincubation with excess IL-18Rα protein (80 μg) eliminated the binding activity of biotinylated IL-18Rα complex proteins (10 μg) almost completely. These results suggest that the sIL-18Rα complex can bind to the surfaces of NK0 cells.

### Soluble interleukin-18 receptor α complex prevents interferon-γ production by a human natural killer cell line

We examined whether the isolated IL-18Rα complex protein was able to prevent or increase the production of IFN-γ by NK0 cells stimulated *in vitro *with IL-2 or IL-18. We reported previously that rhIL-18 proteins synergistically increased the production rhIL-2-induced IFN-γ by NK0 cells [30]. Here we found that the sIL-18Rα complex protein alone did not induce IFN-γ production by NK0 cells and did not synergistically induce IFN-γ production by NK0 cells stimulated with IL-2 alone or IL-18 alone (data not shown). Therefore, we examined whether the sIL-18Rα complex would be able to prevent IFN-γ production by NK0 cells stimulated *in vitro *with IL-2 or IL-18. Interestingly, IFN-γ production by NK0 cells stimulated with IL-2 or IL-18 was dose-dependently inhibited by the sIL-18Rα complex (0.003 to 10 μg/mL; approximately 70% to 80% inhibition at 10 μg/mL) (Figure 2B). Thus, the sIL-18Rα complex exhibited antagonistic, but not agonistic, activity. In addition, treatment with rhIL-18Rα protein (0.1, 1 and 10 μg/mL) inhibited the production of IFN-γ by NK0 cells stimulated with the combination of rhIL-2 (50 IU/mL) and rhIL-18 (50 ng/mL) by 0%, 2% and 34%, respectively (data not shown). These results show that the inhibitory effect of the sIL-18Rα complex on IFN-γ production by NK0 cells stimulated *in vitro *with IL-18 or IL-2 is much stronger than that of rhIL-18Rα protein.

### Increased levels of soluble interleukin-18 receptor α and inflammatory cytokines in sera of rheumatoid arthritis patients

**Figure 3:**
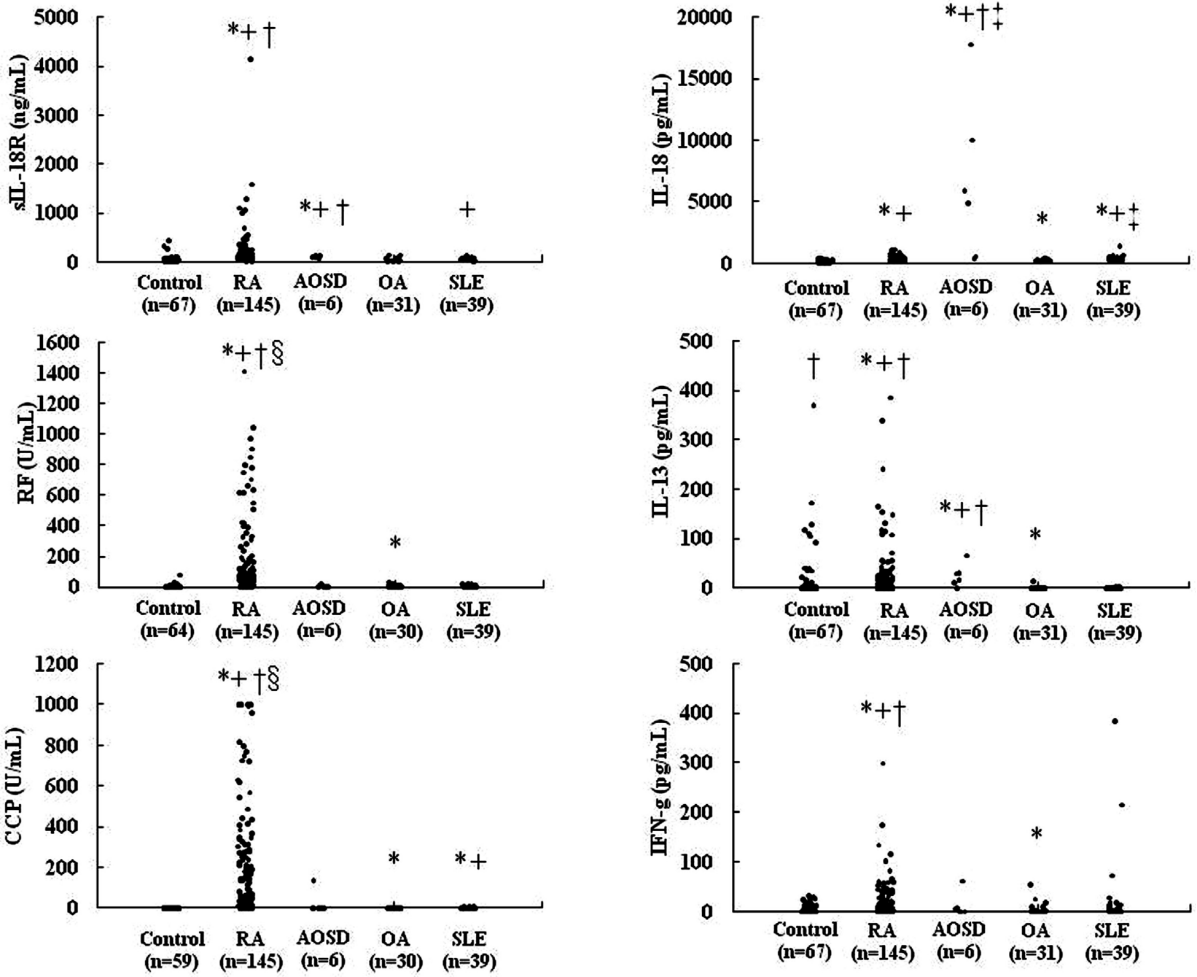
**Levels of soluble interleukin-18 receptor α complex were increased in sera of rheumatoid arthritis and adult-onset Still's disease patients**. IL = interleukin; sIL-18R = soluble IL-18 receptor; AOSD = adult-onset Still's disease; RF, rheumatoid factor; CCP = anticyclic citrullinated peptide; OA = osteoarthritis; SLE = systemic lupus erythematosus. *P < 0.05 vs. healthy controls. ^+^*P *< 0.05 vs. OA. ^†^*P *< 0.05 vs. SLE. ^‡^*P *< 0.05 vs. RA. ^§^*P *< 0.05 vs. AOSD.

We examined whether the levels of the sIL-18Rα complex, IgM RF, anti-CCP antibody, IL-18, IL-13 and IFN-γ were increased in the sera of RA patients and compared to the levels in patients with inflammatory joint disease (adult-onset Still's disease), a noninflammatory arthritic condition (OA) and an autoimmune disease (SLE) (Figure 3). All of the following data are expressed as means ± SEM. The serum levels of sIL-18Rα complex in RA patients (186.0 ± 33.5 ng/mL, *n *= 145) and adult-onset Still's disease (98.2 ± 8.9 ng/mL, *n *= 6) were significantly (*P *< 0.001) higher than those in the healthy controls (52.3 ± 8.5 ng/mL, *n *= 67), OA patients (38.6 ± 5.4 ng/mL, *n *= 31) and SLE patients (44.6 ± 3.2 ng/mL, *n *= 39). The serum levels of sIL-18Rα complex were not significantly different between RA patients and adult-onset Still's disease patients. The serum levels of IgM RF and anti-CCP antibody in the RA patients were also significantly (*P *< 0.0001) higher than in the controls (Table 1). However, the serum level of sIL-18Rα complex was not significantly associated with that of IgM RF and anti-CCP antibody in RA patients. In addition, sIL-18Rα complex was elevated in some RA patients with high serum levels of IgM RF. These results suggest that RF may not influence the detection of sIL-18Rα complex. The serum levels of IL-18 in 67 healthy controls, 145 patients with RA, 6 patients with adult-onset Still's disease, 31 patients with OA and 39 patients with SLE were 159.9 ± 10.9, 299.4 ± 16.1, 6,566.1 ± 2,679.7, 195.5 ± 12.8 and 372.3 ± 32.6 pg/mL, respectively. The serum levels of IL-13 in the same groups were 20.2 ± 6.9, 24.0 ± 4.5, 25.4 ± 9.2, 0.5 ± 0.4 and 0.2 ± 0.1 pg/mL, respectively. The serum levels of IFN-γ in the same groups were 5.4 ± 1.0, 17.3 ± 3.0, 12.2 ± 9.9, 4.1 ± 2.0, and 20.4 ± 11.1 pg/mL, respectively. The serum levels of IL-18, IL-13 and IFN-γ in the RA patients were significantly (*P *< 0.01) higher than in OA and SLE patients as well as in healthy controls. There was no significant association between serum levels of sIL-18Rα complex and that of IFN-γ in RA patients. There was also no significant association between the serum level of sIL-18Rα complex and that of IL-13 or IL-18 in RA patients. In addition, the serum levels of IL-18Rα complex, IL-13 and IFN-γ were not significantly associated with the Steinbrocker functional classification score, the joint damage score, DAS28 (CRP level), DAS28 (ESR), HAQ value, WBC count, CRP level or smoking status in RA patients. These results suggest that the sIL-18Rα complex may not simply be used in the evaluation of joint damage and/or disease activity in RA patients. It is noteworthy that serum levels of IL-18 in patients with adult-onset Still's disease were greatly increased compared to control subjects (Figure 3) as previously reported [18]. The serum levels of IL-18 were significantly associated with HAQ values in RA patients analyzed in this study (data not shown).

**Figure 4:**
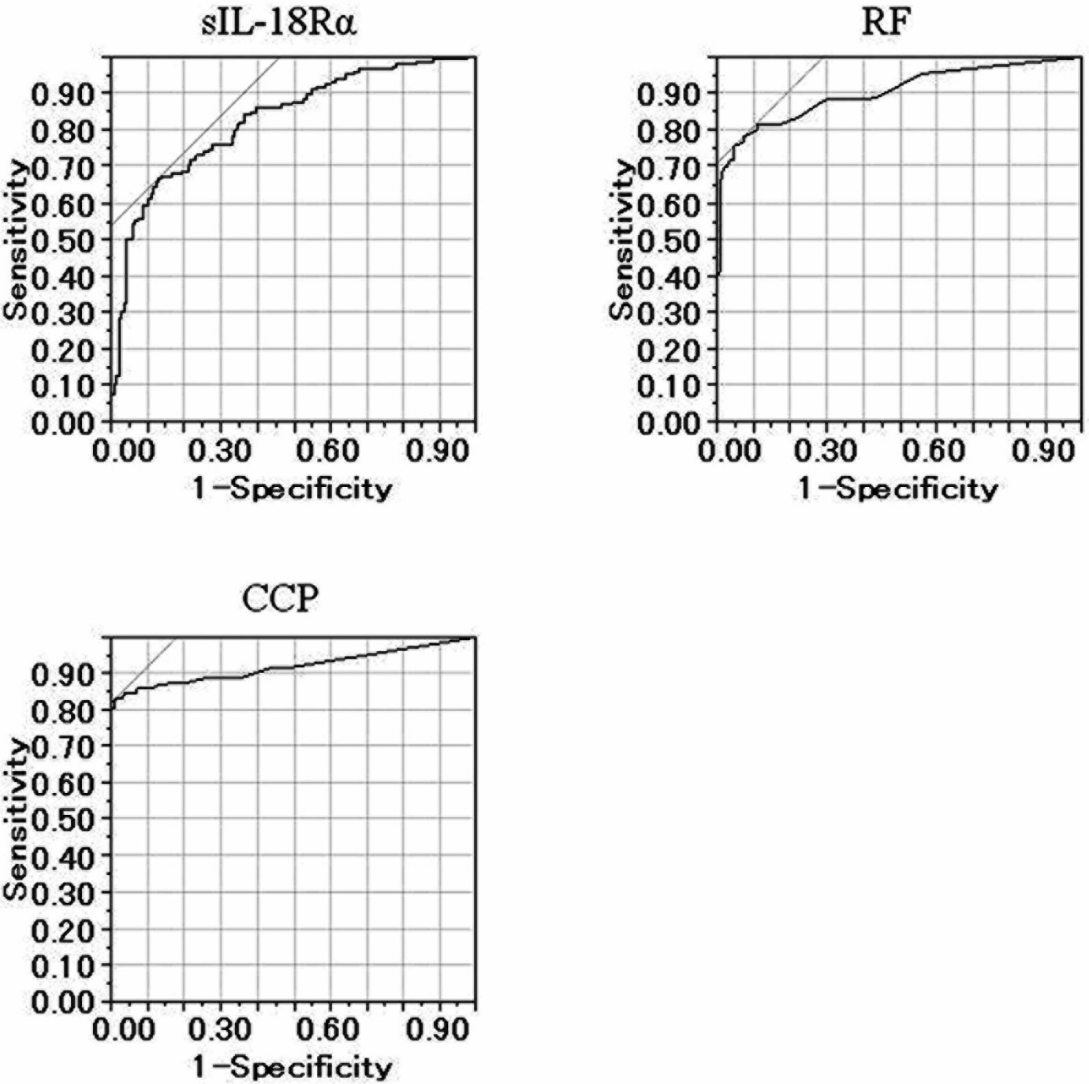
**Area under the receiver operating characteristic curves for detection of rheumatoid arthritis by reference to the level of serum soluble interleukin-18 receptor α complex, immunoglobulin M rheumatoid factor, and anticyclic citrullinated peptide antibody**. The area under the receiver operating characteristic curve (ROC-AUC) for the soluble interleukin-18 receptor α (sIL-18Rα) complex was 0.826. At a cutoff point of 63.1 ng/ml, corresponding to the greatest sum of specificity and sensitivity, the specificity was 0.876 and the sensitivity was 0.662 for detection of rheumatoid arthritis. The ROC-AUC for the serum level of immunoglobulin M rheumatoid factor (RF) was 0.902. At a cutoff point of 19 U/mL, the specificity was 0.955 and the sensitivity was 0.752. The ROC-AUC for the serum level of anticyclic citrullinated peptide (CCP) antibody was 0.921. At a cutoff point of 6.5 U/mL, the specificity was 0.992 and the sensitivity was 0.828.

Using ROC-AUC analysis, we then evaluated whether the serum levels of the IL-18Rα complex, IgM RF, anti-CCP antibody, IL-18, IL-13 and IFN-γ would allow us to discriminate patients with RA from patients with OA or SLE and from healthy controls. The ROC-AUC analysis for the serum level of the IL-18Rα complex was 0.826. At a cutoff point of 63.1 ng/ml, corresponding to the greatest sum of specificity and sensitivity, the specificity was 0.876 and the sensitivity was 0.662 for detection of RA. The ROC-AUC analysis for the serum level of IgM RF was 0.902. At a cutoff point of 19 U/mL, the specificity was 0.955 and the sensitivity was 0.752. The ROC-AUC analysis for the serum level of anti-CCP antibody was 0.921. At a cutoff point of 6.5 U/mL, the specificity was 0.992 and the sensitivity was 0.828 (Figure 4). The ROC-AUC analysis for the serum IL-18 level was 0.626. At a cutoff value of 171.2 pg/mL, the specificity was 0.409 and the sensitivity was 0.835. The ROC-AUC analysis for the serum IL-13 level was 0.740. At a cutoff value of 0.7 pg/mL, the specificity was 0.825 and the sensitivity was 0.676. The ROC-AUC analysis for the serum IFN-γ level was 0.656. At a cutoff value of 1.8 pg/mL, the specificity was 0.620 and the sensitivity was 0.655. Taken together, these data suggest that the serum level of the sIL-18Rα complex is better able to discriminate RA patients than the serum levels of IL-18, IL-13 or IFN-γ.

### Tumor necrosis factor inhibitors decreased levels of soluble interleukin-18 receptor α in sera of rheumatoid arthritis patients

**Figure 5:**
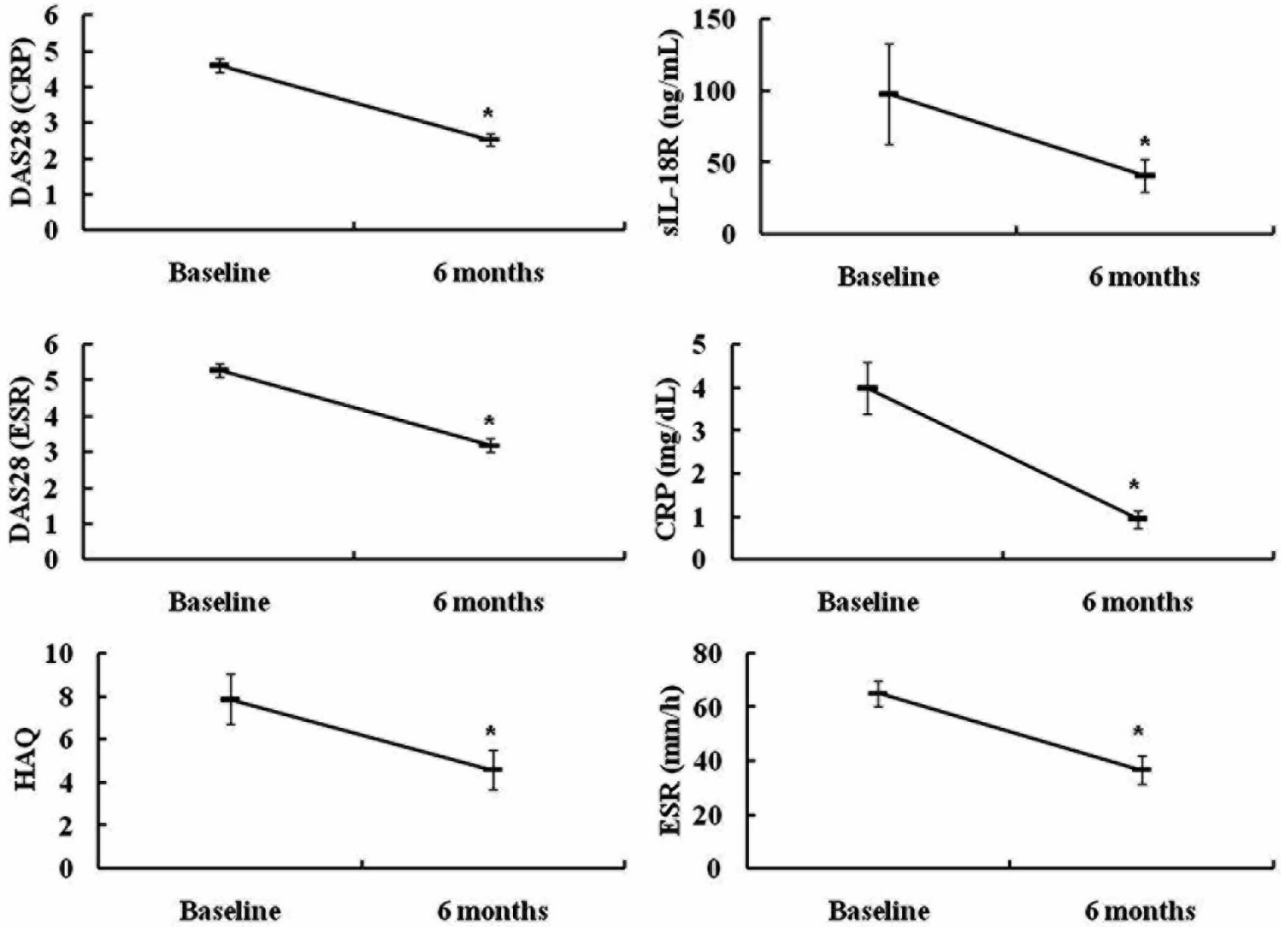
**Tumor necrosis factor inhibitors decreased levels of soluble interleukin-18 receptor α in sera of rheumatoid arthritis patients**. Twenty-nine rheumatoid arthritis patients (two males and twenty-seven females) were treated with the tumor necrosis factor inhibitor etanercept (25 mg once or twice weekly). The 28-joint Disease Activity Score (DAS28) (C-reactive protein (CRP) level), DAS28 (erythrocyte sedimentation rate (ESR)), Health Assessment Questionnaire (HAQ), soluble interleukin-18 receptor α complex in the sera, CRP level and ESR were evaluated before and 6 months after treatment with etanercept. *P < 0.05 vs. baseline.

We treated 29 RA patients (2 males and 27 females; mean age (± SEM), 56.7 ± 2.8 years) with the TNF inhibitor etanercept (25 mg once or twice weekly). Twenty-six (89.7%) or 15 (51.7%) of 29 RA patients were treated with prednisolone (6.27 ± 0.63 mg/day) and/or methotrexate (7.93 ± 0.92 mg/day) in combination with etanercept. We evaluated the serum levels of sIL-18Rα, CRP, ESR, DAS28 (CRP), DAS28 (ESR) and HAQ score before and 6 months after treatment with etanercept. We observed significant (*P *< 0.0001) improvements in the serum levels of sIL-18Rα complex, CRP, ESR, DAS28 (CRP), DAS28 (ESR) and HAQ score in the 29 RA patients (Figure 5).

## Discussion

In this study, we have demonstrated the presence of the sIL-18Rα chain in human serum. The sIL-18Rα complex is composed of the soluble forms of the IL-18Rα and IL-18Rβ chains and IL-18. The complex was shown to bind to the surfaces of the human NK cell line NK0 and prevented the production of IFN-γ by NK0 stimulated with IL-18 and IL-2 *in vitro*. Thus our results suggest that the sIL-18Rα complex binds to the cell surface and attenuates IL-18 signaling.

A previous study showed that the molecular mass of IL-18Rα protein isolated from the Hodgkin's disease cell line L428 was 64 to 100 kDa. The DNA sequence of hIL-18Rα encodes the signal peptide (Met 1 to Ala 19) domains 1 through 3, to which IL-18 can bind (Glu 20 to Arg 329), and the transmembrane domain (Gly 330 to Tyr 351) [13]. The predicted molecular mass of the extracellular domain of the hIL-18Rα chain is approximately 36 kDa. These results suggest that hIL-18Rα protein on cell surfaces is highly glycosylated and shows sugar chain heterogeneity [13]. Our SDS-PAGE analysis using CBB staining showed that the sIL-18Rα complex comprised a 50-kDa major band and other bands of approximately 60 to 80, 30 and 15 kDa. Western blot analysis showed that the IL-18Rα protein associated with the IL-18Rα complex had at least two components of approximately 50 and 30 kDa. As our anti-IL-18Rα mAb (H44) recognizes the extracellular domain of hIL-18Rα protein [30], the IL-18Rα protein associated with the IL-18Rα complex would have been derived from the extracellular domain of IL-18Rα. However, the molecular mass of the sIL-18Rα protein associated with the IL-18Rα complex was much smaller than that of the extracellular domain of hIL-18Rα as reported previously on the cell surface [13,30]. Therefore, the sIL-18Rα protein appears to be glycosylated or shows heterogeneity of its sugar chains.

Soluble cytokine receptors of the IL-1R/Toll-like receptor superfamily are thought to be generated by intramembrane proteolysis and/or alternative splicing. sIL-1RI is generated by the action of metalloprotease [5]. sIL-1RII can be generated by both proteolytic cleavage of receptor ectodomains and alternative splicing events. β-secretase 1 and β-secretase 2 can function as IL-1RII sheddases that cleave the IL-1RII ectodomain at a site adjacent to the α-secretase site [2,36]. TNF-α-converting enzyme (ADAM17), a transmembrane metalloprotease, is also responsible for the proteolytic release or shedding of IL-1RII [37]. It has been reported that sIL-1RAcP is generated by alternative splicing rather than by ectodomain cleavage [38]. Therefore, the sIL-18Rα chain seems to be generated by intramembrane proteolysis and/or alternative splicing events.

IL-18Rβ shares structural similarities with IL-1RAcP [39]. Like sIL-1RAcP, a short form of the IL-18Rβ (sIL-18Rβ mRNA transcript has been described in the rat [40], as well as in human and mouse [39]. A recent study showed that intravenous administration of adenoviruses encoding sIL-18Rβ promoted collagen-induced arthritis in DBA/1 mice [39]. However, the biological function of sIL-18Rβ is largely unknown. In the present study, we have shown that the sIL-18Rα complex comprises IL-18 and sIL-18Rα and sIL-18Rβ. Two different anti-IL-18Rβ mAb clones, 132016 and 132029 (R&D Systems, Inc.), detected bands of approximately 60, 40 and 18 kDa in the isolated sIL-18Rα complex (data not shown). These two anti-IL-18Rβ mAbs recognize the extracellular domain of the hIL-18Rβ chain. Therefore, sIL-18Rβ in human serum is likely derived from the extracellular domain of the hIL-18Rβ chain generated by differential messenger RNA splicing.

It has been reported that the levels of various inflammatory cytokines and chemokines, such as IL-2, IL-4, IL-5, IL-6, IL-7, IL-10, IL-13, IFN-γ, granulocyte colony-stimulating factor, granulocyte macrophage colony-stimulating factor, monocyte chemotactic protein-1 and macrophage inflammatory protein-1β, are increased in the sera of RA patients [41]. Inflammatory cytokines and chemokines, including IL-18, IL-13 and IFN-γ, may play an important role in the pathogenesis and development of RA [8,9]. In this study, we have shown that the serum levels of the IL-18Rα complex in patients with RA or adult-onset Still's disease were significantly higher than those in healthy controls as well as in patients with OA or SLE. However, the origin of the IL-18Rα complex is still unclear. Therefore, we analyzed the levels of the sIL-18Rα complex in both the synovial fluid and sera in three RA patients (26-, 39- and 81-year-old females). The levels of sIL-18Rα complex in the synovial fluid in these RA patients were 22.1, 30.8 and 6.9 ng/mL, respectively. The levels of sIL-18Rα complex in their sera were 39.8, 23.6 and 16.2 ng/mL, respectively. Thus, the levels of sIL-18Rα complex were not greatly increased in the synovial fluid in these three RA patients. Further analysis is needed to address the origin of the IL-18Rα complex in RA patients.

ROC-AUC analysis revealed that RA patients could be discriminated by the serum level of the sIL-18Rα complex as determined by ELISA. Moreover, the TNF inhibitor etanercept significantly decreased levels of sIL-18Rα in the sera of 29 RA patients 6 months after the treatment. Thus, the sIL-18Rα complex may play an important role in the inflammatory process of RA and adult-onset Still's disease. Our present results suggest that the level of the sIL-18Rα complex in serum has potential clinical application as a biomarker for the diagnosis or differential diagnosis of RA or for the evaluation of disease activity in RA patients treated with TNF inhibitors.

## Conclusions

In this study, we evaluated the levels and characteristics of the sIL-18Rα complex in the sera of patients with RA and compared these to control populations. Our study indicates that sIL-18Rα is present in the sera of RA patients and is complexed with IL-18 and IL-18Rβ. The levels detected by ELISA were substantially higher in the RA patients and patients with adult-onset Still's disease than in controls, and modeling using ROC-AUC analysis suggested that this assay might be of diagnostic value.

## Abbreviations

BP: binding protein; CBB: Coomassie Brilliant Blue; IL: interleukin; IL-1RacP: IL-1 receptor accessory protein; IRA: interleukin 1-associated kinase; OA: osteoarthritis; PAGE: polyacrylamide gel electrophoresis; RA: rheumatoid arthritis; RF: rheumatoid factor; ROC-AUC: area under the receiver operating characteristic curve; SLE: systemic lupus erythematosus; TNF: tumor necrosis factor.

## Competing interests

TH holds a Japanese patent relating to sIL-18Rα complex (no. 4257946). All other authors declare that they have no competing interests.

## Authors' contributions

ST and TH performed the experiments, and performed the data analysis and data interpretation. ST, TH and HA drafted the manuscript. KM, YS, HO, ST, HI, TK, SH, HI and TF collected samples. All authors read and approved the final manuscript.
